# Modulation of visceral pain by brain nuclei and brain circuits and the role of acupuncture: a narrative review

**DOI:** 10.3389/fnins.2023.1243232

**Published:** 2023-11-01

**Authors:** Zhiqiang Dou, Na Su, Ziyang Zhou, Aoyue Mi, Luyao Xu, Jiazheng Zhou, Sizhe Sun, Yanyi Liu, Mingyao Hao, Zhaofeng Li

**Affiliations:** ^1^College of Acupuncture and Moxibustion and Tuina, Shandong University of Traditional Chinese Medicine, Ji’nan, China; ^2^First Clinical Medicine College, Shandong University of Traditional Chinese Medicine, Ji’nan, China; ^3^External Treatment Center of Traditional Chinese Medicine, Affiliated Hospital of Shandong University of Traditional Chinese Medicine, Ji’nan, China; ^4^International Office, Shandong University of Traditional Chinese Medicine, Ji’nan, China

**Keywords:** visceral pain, brain nuclei, brain circuit, acupuncture, analgesia

## Abstract

Visceral pain is a complex and heterogeneous pain condition that is often associated with pain-related negative emotional states, including anxiety and depression, and can exert serious effects on a patient’s physical and mental health. According to modeling stimulation protocols, the current animal models of visceral pain mainly include the mechanical dilatation model, the ischemic model, and the inflammatory model. Acupuncture can exert analgesic effects by integrating and interacting input signals from acupuncture points and the sites of pain in the central nervous system. The brain nuclei involved in regulating visceral pain mainly include the nucleus of the solitary tract, parabrachial nucleus (PBN), locus coeruleus (LC), rostral ventromedial medulla (RVM), anterior cingulate cortex (ACC), paraventricular nucleus (PVN), and the amygdala. The neural circuits involved are PBN-amygdala, LC-RVM, amygdala-insula, ACC-amygdala, claustrum-ACC, bed nucleus of the stria terminalis-PVN and the PVN-ventral lateral septum circuit. Signals generated by acupuncture can modulate the central structures and interconnected neural circuits of multiple brain regions, including the medulla oblongata, cerebral cortex, thalamus, and hypothalamus. This analgesic process also involves the participation of various neurotransmitters and/or receptors, such as 5-hydroxytryptamine, glutamate, and enkephalin. In addition, acupuncture can regulate visceral pain by influencing functional connections between different brain regions and regulating glucose metabolism. However, there are still some limitations in the research efforts focusing on the specific brain mechanisms associated with the effects of acupuncture on the alleviation of visceral pain. Further animal experiments and clinical studies are now needed to improve our understanding of this area.

## 1. Introduction

Visceral pain is a nociceptive phenomenon originating from the thoracic or abdominal visceral organs that is distinct from general somatic pain and is caused predominantly by mechanical pulling, spasm, ischemia, or inflammation of the internal organs. The characteristics of visceral pain include an unclear etiology, diffused pain, and chronic overlapping pain caused by the involvement of multiple organs (Grundy et al., 2019). Visceral pain is one of the most common reasons driving patients to seek medical help (Pusceddu and Gareau, 2018). Epidemiological analysis has shown that up to 20% of adults suffer from chronic visceral pain worldwide. However, visceral pain is not only limited to chronic pain; it can also occur in acute episodes of pain, such as peptic/intestinal ulcers, cholecystitis, and appendicitis, as well as functional abdominal pain, pancreatitis, and some forms of chronic intestinal pain. Moreover, in addition to pain sensation, patients with visceral pain, especially those with chronic pain, often experience a variety of psychological and emotional problems, including depression, anxiety, fear, insomnia, and cognitive impairment. These adverse effects can further exacerbate pain to form a vicious cycle that can seriously affect a patient’s quality of life and increase the social and medical burden (Arango-Dávila and Rincón-Hoyos, 2018; Clauw et al., 2019; Michaelides and Zis, 2019; Woelk et al., 2020; Todd et al., 2022; Bäckryd and Alföldi, 2023). Therefore, the etiology, pathogenesis, and treatment of visceral pain have always been a hot topic and focus of scientists.

The pathogenesis of visceral pain is complex and involves a series of processes from the microbial level to the brain (Grundy et al., 2019). Previous studies have mainly focused on the peripheral nervous system and the spinal cord. In recent years, with the rise of neuroscience, the rich accumulation of knowledge related to neurobiology, neuromedicine, and the continuous development of various technologies such as neural tract tracking, photogenetics, chemical genetics, and functional magnetic resonance imaging, research on the central transmission and regulatory mechanisms of visceral pain has become increasingly important. For example, more and more studies have noticed that many brain regions and neural circuits mediate visceral pain. However, the relevant literature is relatively scattered and the precise mechanisms and interactions involved have yet to be fully elucidated; there is a need for further investigations, especially using integrative approaches.

The current clinical treatment of visceral pain is mainly based on conventional medications, such as oral non-steroidal anti-inflammatory drugs and opioids. However, these drugs are associated with certain side effects; in addition, these drugs can be ineffective if the processing of pain in the central nervous system pain has been altered (Bouwense et al., 2015). Therefore, there is an urgent need to develop new analgesic therapies Acupuncture has unique advantages for analgesia due to its efficacy and safety. Clinical practice has provided evidence to support the fact that acupuncture can relieve visceral pain (Lund and Lundeberg, 2016; Juel et al., 2017; Pei et al., 2018); it is possible that the mechanisms involved includes alterations in pain-related brain regions and the activities of certain neural circuits.

In this article, based on various animal models of visceral pain, we describe the brain nuclei and neural circuits that regulate visceral pain. Summarizing the brain mechanism of acupuncture regulating visceral pain, we found that the effect of acupuncture on visceral pain depends on the interactions with the supraspinal central structures. Among them, multiple neurotransmitters and/or receptors are involved in this acupuncture-related analgesic process. In addition, acupuncture can also regulate visceral pain by affecting functional connectivity and regulating glucose metabolism in specific brain regions. With the continuous development of functional magnetic resonance imaging (fMRI) and calcium imaging techniques, further basic and clinical research can be carried out in the future, constantly searching for new brain nuclei and neural circuits and providing new targets or treatment strategies for clinical treatment of visceral pain and identify specific acupuncture-related pathways and mechanisms.

## 2. Animal models of visceral pain

The basic research on visceral pain has made rapid progress, but its clinical transformation and application are complicated, so it is necessary to conduct animal research. A reasonable visceral pain model should comply with the theoretical conditions of disease occurrence and be able to correspond to its specific physiological and pathological characteristics. Visceral pain is caused by mechanical traction, ischemia, or inflammation of the internal organs. Several models can be used to investigate these different forms of stimulation, including the mechanical dilated visceral pain model, the ischemic visceral pain model, the inflammatory visceral pain model, and the neonatal stage model. The mechanical dilation visceral pain model predominantly induces visceral pain in hollow visceral organs via balloon dilation, including the gastric/duodenal dilation colorectal and ureteral dilation models. The ischemic visceral pain model causes pathological pain via vascular occlusion, especially arterial occlusion; at present, this type of model is dominated by the coronary artery occlusion model. Inflammatory visceral pain models involve inflammatory pain caused by the injection of stimulatory drugs into the organs of animal models, including torsion test models, and models of pericarditis, sigmoiditis, ulcerative colitis and cystitis. Moreover, the commonly used models of visceral pain also include maternal–infant separation models and stress models.

## 3. Brain nuclei and circuits related to visceral pain

Many brain nuclei are involved in the central transmission and modulation of visceral pain. Different brain nuclei and their projections form neural circuits that regulate the perception of visceral pain and negative emotions. Therefore, understanding the specific brain nuclei and circuits underlying visceral pain and improving the central neuromodulation mechanism may provide suitable targets for the clinical treatment of visceral pain.

### 3.1. The nucleus of the solitary tract

The nucleus of the solitary tract (NTS) is located on the dorsal side of the medulla oblongata and the lateral side of the vagus nerve, serving as the top of the solitary tract. This tract forms a Y-shaped column of cells in the rostral direction and runs the entire length of the medulla oblongata. As the central visceral sensory nucleus in the brainstem, the NTS receives projections from the vagus nerve that transmits visceral sensations from the chest and abdomen, which participate in the transmission and regulation of visceral pain information (Graven-Nielsen et al., 1998; Emch et al., 2000). Research has shown that c-Fos serves as a cellular marker for neuronal activity and represents an efficient indicator for evaluating the degree of nociceptive stimuli. Different forms of visceral nociceptive stimuli can induce high expression levels of c-Fos in the NTS. In the rat model of visceral pain induced by the intraperitoneal injection of acetic acid, the expression levels of c-Fos protein and GFAP in the NTS of the medulla oblongata increased, thus indicating that both astrocytes and neurons in the NTS are involved in the regulation of visceral pain (Qin et al., 2006). These findings were consistent with data reported by Bonaz et al. (2000) who observed c-Fos labeling in the NTS of rats with visceral pain induced by acetic acid injection. Following gastric dilation in rats, the expression of c-Fos in the NTS mediated by the vagus nerve increased; furthermore, the c-Fos immune response was significantly weakened after vagotomy. These data indicated that parasympathetic inputs help to handle harmful visceral stimuli, possibly by promoting the emotional components of visceral pain (Traub et al., 1996). Two weeks after ligation of the left anterior descending coronary artery, synaptophysin immunoreactive points and c-Fos immunoreactive neurons in the rat NTS increased significantly while excitatory synaptic transmission was enhanced. Furthermore, specific behavioral traits associated with visceral pain were significantly increased in a rat model of CMI (Li et al., 2015). In a previous study, Bai et al. (2019) investigated the regulatory role of NTS in the visceral hypersensitivity response of rats with chronic pancreatitis induced by drug and chemical genetic methods. These authors found that the injection of 2,4,6-trinitrobenzene sulfonic acid (TNBS) significantly increased the number of neurons expressing c-Fos in the caudal region of NTS, thus suggesting that the caudal NTS is a key center for processing incoming signals in pancreatitis. The enhancement of excitatory synaptic transmission in the NTS contributes to the visceral hypersensitivity response in rats with pancreatitis. Collectively, these studies indicate that the NTS plays an important role in regulating visceral pain.

### 3.2. The parabrachial nucleus

The parabrachial nucleus (PBN) is located in the dorsolateral aspect of the pons and is divided into three main subnuclei: the medial parabrachial nucleus, the lateral parabrachial nucleus (LPB) and the ventral Kölliker-Fuse nucleus. It is considered a relay station for the spinal cord to transmit pain and aversion information to the brain. Electrophysiological recordings have demonstrated that somatic and visceral pain can activate neurons in the lateral PBN (Gauriau and Bernard, 2002). Similarly, previous researchers measured the induction of c-Fos in the PBN in response to various threats and found that the induction of c-Fos in the lateral region of the PBN was consistently associated with noxious somatosensory and visceral injury. This data suggested that the PBN is extensively involved in encoding and transmitting information associated with visceral pain at both the sensory and affective levels. In another study, researchers activated the projection of the spinal VGLUT3 neurons to the PBN; this induced aversion in mice with gastrointestinal inflammation but did not affect visceromotor responses (VMRs). This data suggested that spinal neurons project emotional but not nociceptive information relating to visceral inflammatory pain to the PBN. In other words, different spinal cord substrates transmit the nociceptive and emotional dimensions of visceral sensory information, respectively (Qi et al., 2023). In addition, studies have observed that there are obvious gender differences in the involvement of visceral nociceptive stimuli in the spinal cord-PBN circuit. For example, the expression of c-Fos in male rats was significantly higher than that in females, and morphine was shown to reduce the c-Fos expression selectively; this may be the factor that causes clinical differences in sensitivity to visceral pain (Murphy et al., 2009).

As a relay station that transmits visceral pain information, the PBN receives projections from the spinal cord and projects this information to the central amygdala (CeA), particularly the paraspinal amygdala pathway (Gauriau and Bernard, 2002). Neurons from the PBN neurons project onto CeA neurons to induce the expression of high levels of calcitonin-generating peptide (CGRP) (Schwaber et al., 1988); this effect is known to be associated with visceral discomfort, aversion, and pain (Palmiter, 2018). In the case of neuropathic pain, LPB CGRP neurons can transmit pain information to CeA neurons via axonal collateral branches (Liang et al., 2016). Similarly, in rats with colitis, enhanced synaptic transmission and increased excitability of CeA neurons were observed at the nociceptive LPB-CeA synapse following electrical stimulation of the LPB, thus indicating the critical role of the LPB-CeA in visceral pain (Han and Neugebauer, 2004). In a mouse model of bladder pain, CGRP in the PBN caused a significant increase in the excitability of CeA neurons. In addition, CGRP signals from the PBN in the CeA exert are known to exert a significant lateral regulatory effect on bladder pain. The administration of CGRP to the right CeA has been shown to increase the behavioral signs of bladder pain while reducing bladder pain-like behavior on the left. These data indicate that the PBN-CeA circuit driven by CGRP neurons can determine the hemispheric lateralization of visceral pain (Allen et al., 2023).

### 3.3. The rostral ventromedial medulla

The rostral ventromedial medulla (RVM) is a key relay station of the downstream pain modulation system. It plays a key role in the central processing of visceral pain, such as bladder distension (Randich et al., 2008), pancreatic pain (Vera-Portocarrero et al., 2006), and visceral hypersensitivity induced by colonic chemical stimuli (Kang et al., 2013). For example, in capsaicin-induced visceral injury perception and associated hypersensitivity rats, there is an enhanced response to colorectal distension (CRD) and persistent on-like spontaneous activity of RVM neurons, suggesting that noxious visceral stimuli may lead to hypersensitivity by promoting long-term sensitization of RVM on-like cells (Sanoja et al., 2010).

### 3.4. The locus coeruleus

In the medulla oblongata, there are two structures that play important roles in the descending inhibitory system: the RVM and the locus coeruleus (LC). Norepinephrine is the primary neurotransmitter that regulates many behaviors, including sleep and wakefulness, the attention stress response, and pain (Benarroch, 2018). Traditionally, the LC has been regarded as a pain suppressive center, relieving visceral pain caused by nociceptive CRD by directly projecting downwards to the spinal cord (Liu et al., 2008; Tsuruoka et al., 2010). However, research has suggested that the balance of functionality in the LC shifts from the inhibition of pain to the promotion of pain (Taylor and Westlund, 2017), thus mediating visceral pain by projecting upward fibers to other brain regions.

The RVM and the LC are nuclei that play critical roles in the downstream pain modulation system (Imbe et al., 2004). Based on functional connectivity (FC) between the LC and RVM, it was possible to determine the direct circuit projection from norepinephrine neurons in the LC to the RVM. Specific excitation of the LC^NA^-RVM circuit was shown to significantly induce hyperalgesia in the colonic viscera of normal mice. Chronic restraint stress models induce anxiety-related psychiatric disorders and colorectal visceral hypersensitivity, conditions that are known as pathological stressed hypersensitivity (Holschneider et al., 2016). The LC^NA^-RVM circuit is activated in both the DSS-induced visceral pain model and the chronic restraint stress-induced visceral hypersensitivity model; the inhibition of this circuit reverses visceral pain or visceral hypersensitivity. Research has suggested that the LC-RVM circuit may be a critical factor in colorectal visceral pain and stress-related psychiatric disorders (Kong et al., 2023). In summary, the specific role of the LC-RVM circuit in visceral pain has been determined by the establishment of models of visceral pain induced in different manners and by utilizing various operational methods such as retrograde labeling and projection-specific chemical genetics.

### 3.5. The amygdala

The amygdala, also known as the amygdala complex, is located in the dorsomedial part of the brain’s temporal lobe. The amygdala is an important structure of the limbic system and contains multiple nuclei of varying sizes, such as the basolateral nucleus group, central nucleus, and cortical nucleus (Šimić et al., 2021). Of these, research has mostly focused on the CeA and basolateral amygdala (BLA), especially the CeA, which is known to play a key role in the coding and modulation of pain and related emotions (Veinante et al., 2013). Research has shown that the neural plasticity of the CeA is positively correlated with visceral pain behavior and drives such behavior by increasing the excitatory transmission and excitability of CeA neurons (Ji et al., 2015). The CeA has also been shown to be necessary for acute stress-induced bladder hyperalgesia in models of visceral pain. The activation of metabotropic glutamate receptors can induce bladder pain sensitization by increasing CeA output (Crock et al., 2012); furthermore, bilateral CeA injury can prevent such hyperalgesia (Deberry et al., 2015). Epigenetic remodeling of the CeA is also known to be a contributing factor to visceral hypersensitivity. Early stress-induced enhanced histone acetylation in the amygdala has been shown to lead to visceral hypersensitivity and anxiety-like behavior in rats. Reversing abnormal epigenetic mechanisms can alleviate chronic symptoms in stressed rats (Louwies et al., 2021; Orock et al., 2021; Guan et al., 2022). In addition, chronic psychological stress has been shown to induce microglial remodeling of the CeA and promote the development of visceral hypersensitivity via synaptic remodeling and the release of inflammatory cytokines (Yuan et al., 2020), which can alleviate visceral pain by regulating microglial activity in the CeA (Yuan et al., 2022).

The insular cortex is located deep in the medial sulcus of the brain and plays an extremely important role in sensory and emotional aspects. The insular cortex is considered to represent a key area for visceral pain (Nagai et al., 2007; Uddin et al., 2017). Increased visceral sensitivity is known to be significantly associated with reduced gray matter volume in the insula and amygdala (Elsenbruch et al., 2014). The comparison of FC changes between healthy and Crohn’s disease patients by magnetic resonance imaging further showed that the progression of Crohn’s disease was negatively correlated with the amygdala-insula circuit, thus indicating a specific functional connection between the amygdala and the insula; this may represent a new mechanism for the treatment of visceral pain (Fan et al., 2020), although further experiments are still needed to verify this contention.

### 3.6. The anterior cingulate cortex

The anterior cingulate cortex (ACC) is an area of the brain that plays a role in visceral stimulation processing. Changes in the activation or deactivation of the ACC can ultimately amplify pain perception. In patients with irritable bowel syndrome, local cerebral blood flow in areas such as the anterior cingulate gyrus, insula, and prefrontal cortex (PFC), increases significantly, with a stronger correlation between activation of the anterior cingulate gyrus and the subjective discomfort of visceral pain (Berman et al., 2000). Human brain imaging studies have shown that IBS patients exhibit more significant ACC activation during painful rectal dilation when compared to healthy subjects (Chang, 2005), thus indicating that the ACC plays a key role in mediating visceral pain, a fact that has been further validated in relevant animal experiments (Tsushima et al., 2021). In visceral hypersensitive rats, the activation of ACC neurons was significantly enhanced (Cao et al., 2008). Similarly, in a rat model of chronic pancreatitis, the presynaptic release of glutamate from pyramidal neurons in the ACC was increased, and the expression of postsynaptic glutamate receptors and phosphorylation was also increased, thus indicating the central sensitization of pain states. A significant analgesic effect can be produced by inhibiting the excitability of pyramidal neurons in the ACC in a manner that was equivalent to the effect of local opioid administration (Ren et al., 2022).

Pain includes two dimensions: pain sensation and emotional experience (Iqbal et al., 2023). Activating the ACC can also regulate pain-related anxiety in mice suffering from acetic acid-induced visceral pain (Zhong et al., 2012). In the DSS mouse model, the firing frequency of ACC glutamate neurons was significantly increased while the inhibition of ACC glutamate neurons alleviated visceral hypersensitivity but did not alleviate depressive-like behavior. Activating ACC glutamate is known to exacerbate visceral pain in mice but significantly improve depressive symptoms (Wu et al., 2023). In addition, the ACC also plays a crucial role in mediating aversion consolidation and memory formation related to visceral nociceptive stimuli (Yan et al., 2012; Iqbal et al., 2022). These studies indicate that the ACC plays a major role in visceral pain sensation and pain emotion, thus paving the way for further research on the mechanisms of ACC-related circuits involved in visceral pain sensation, emotion, perception. However, the ACC exhibits heterogeneity in mediating negative pain emotions; this may be related to different types of neurons and pain models.

The ACC can accept a wide range of projections from the LC and participate in a range of behaviors such as wakefulness, attention, cognitive perception, pain, and itching responses (Gompf et al., 2010; Krebs et al., 2013; Koga et al., 2020; Hallock et al., 2023). A recent study found that the LC-ACC neural circuit can precisely regulate the establishment and consolidation of pain aversion memory in rats. The specific activation or inhibition of LC neurons projecting to the ACC was shown to significantly enhance or reduce pain aversion memory in rats (Iqbal et al., 2023). Previous research on pain and related emotions has focused predominantly on neurons and largely neglected the influence and regulation of astrocytes on neurons. Astrocytes metabolize lactic acid via glycolysis to provide energy for neuronal cells and to release neurotransmitters such as glutamate as the third synapse. Astrocytes regulate nociceptive synaptic transmission and network functionality via the interaction of neuron neurotransmitters and glia, thus playing an essential role in different pathological conditions, including chronic pain and emotional memory (Lu and Gao, 2023). Based on this, Iqbal et al. (2023) discovered that astrocytes in the ACC are vital targets for the formation of pain aversion memory. Inhibiting astrocytes in the ACC not only effectively reduced pain aversion memory in rats but also counteracted the memory-enhancing effect of photogenetic activation of LC-ACC projection neurons.

Visceral pain includes emotional and cognitive signs besides sensory components, among which decision-making is a fundamental cognitive process for adaptation. In a state of visceral hypersensitivity, rats often exhibit a reduction in decision-making ability and neuronal dysfunction between the BLA and ACC, such as reduced long-term synaptic enhancement and consistent damage to the spike field (Cao et al., 2016; Chen et al., 2018). These data led to the speculation that this may be the leading cause of decision-making deficits in a state of chronic visceral pain. Hasan et al. (2023) further found that the reduction in decision-making ability related to VH may be closely related to a reduction of myelination in the ACC region and is caused by disruption of synchronization in neural activity in the ACC-BLA. By locally administering ACC myelinating drugs or by activating ACC astrocytes through chemical genetics to restore myelination in the brain area, the ACC-BLA synchronicity can be repaired, thereby improving the decision-making ability of a rat model of IBS. Collectively, these results indicate that the ACC-BLA circuit mediates participation in decision-making disorders caused by visceral pain; consequently, this circuit is an important target for the treatment of cognitive disorders related to chronic visceral pain.

The claustrum (CL) is a thin layer of gray matter located beneath the insular cortex, of the brain which appears to be interconnected with various regions of the cortex (Jackson et al., 2020; Smith et al., 2020) and receives many fiber inputs from the frontal cortex region, including the orbitofrontal cortex, prefrontal cortex, and the ACC (Zingg et al., 2018). Previous research discovered a neural circuit relationship between the ACC and CL and showed that inhibiting the activity of this circuit could acutely alleviate inflammatory abnormal pain (Ntamati et al., 2023). However, another study suggested that glutamate neurons in the CL projected to the ACC to participate in mediating visceral pain; however, these neurons were not closely related to inflammatory pain. In a chronic visceral pain model established by neonatal maternal deprivation, glutamate neurons in both the CL and ACC could mediate visceral pain responses. Moreover, virus tracing demonstrated that the ACC receives information input from the CL; intervention of the projections of glutamate neurons from the CL to the ACC caused significant changes in the visceral pain behavior of mice, thus suggesting that the glutamate projections of the CL-ACC can synergistically regulate visceral pain. To investigate the specificity of the CL-ACC neural circuit, injections of complete Freund’s into the soles of experimental mice did not alter the excitability of neurons in the CL brain area; furthermore, photogenetic regulation of the CL-ACC neural circuit did not alter the inflammatory pain response of these mice. Collectively, these results suggest that the CL-ACC may be a specific neural circuit that regulates visceral pain rather than inflammatory surface pain (Xu Q. Y. et al., 2022).

### 3.7. The paraventricular nucleus of hypothalamus

The paraventricular nucleus of hypothalamus (PVN) is located on both sides of the superior part of the hypothalamus in the third ventricle. This is one of the most prominent nuclei in the anterior hypothalamus. The expression levels of c-Fos in the PVN of a mouse model of visceral pain induced by acetic acid have been shown to increase; furthermore, inhibiting the activity of neurons in the PVN can alleviate acute acetic acid-induced visceral pain in mice (Xu J. N. et al., 2022). Corticotropin-releasing hormone (CRH) is a peptide hormone and neurotransmitter that is synthesized and localized widely in the hypothalamus. Previous research confirmed that the activation of CRH neurons in the PVN contributes to visceral hypersensitivity in mice (Zhang et al., 2016). Previous research involving a rat model of visceral hypersensitivity response induced by neonatal colorectal dilation showed that PVN CRH neurons were activated in adulthood and that the administration of exogenous GABA and GABA A receptor agonists could eliminate visceral hyperalgesia (Song et al., 2020). Studies have utilized the activity of CRH neurons in the PVN to differentiate between visceral pain-sensitive and non-susceptible mice under different pathological and physiological states in response to visceral stimuli. Analysis showed that susceptible mice exhibited increased expression levels of CRH genes and proteins in the PVN, significantly increased discharge frequency in the CRH neurons, and enhanced neuronal excitability (Huang et al., 2021). In summary, the enhanced excitability of the PVN CRH neurons can mediate the occurrence and development of visceral pain.

It is known that the excitability of CRH neurons is significantly enhanced in PVN when induced by different methods in mice with visceral hypersensitivity. Further analysis revealed that the projection neurons in the anterior ventral region of thebed nucleus of the stria terminalis (avBNST) successfully formed functional synapses with PVN CRH neurons. Researchers previously evaluated visceral hypersensitivity and divided mice undergoing maternal–infant separation into two subgroups (visceral pain-sensitive and non-susceptible) and demonstrated a significant increase in the excitability of CRH neurons in the PVN of susceptible mice. These researchers also verified that the avBNST region can project glutamate and GABAergic neurons to the PVN CRH neurons, respectively. The firing frequency of GABAergic projection neurons in the avBNST, which controls PVN in susceptible mice, was significantly reduced, while the firing frequency of glutamatergic projection neurons was significantly increased. Activation of GABAergic projection neurons in the avBNST-PVN loop by light/genetics, or by the inhibition of glutamatergic neurons, was shown to increase the threshold to visceral pain in susceptible mice, further confirming that the activation of glutamatergic projections and the inhibition of GABAergic projections in avBNST-PVN neurons jointly mediate the occurrence and development of chronic visceral pain in susceptible mice (Huang et al., 2021). Another study found that most GABAergic neurons in the PVN project to the avBNST and that their excitability decreases during the visceral hypersensitivity response. Furthermore, activating the PVN-avBNST GABAergic neurons alleviated the visceral hypersensitivity response in neonatal CRD rats, while de-inhibition facilitated the excitation of CRH neurons, thereby mediating the visceral hypersensitivity response (Song et al., 2020).

The lateral septum (LS) is the dorsal part of the septum, which is divided into three parts: the dorsal, middle and ventral lateral septum (LSV) (Risold and Swanson, 1997). The LS is involved in the regulation of emotion and a variety of behaviors. In a rat model of visceral pain, local cerebral blood flow in the LS was significantly enhanced, thus suggesting that the LS may be involved in visceral hyperalgesia. Researchers found that the activity of LSV neurons was enhanced when mice were exposed to CRD. In contrast, LSV neurons did not change significantly when mice were stimulated on the body surface. Viral tracing further revealed that visceral pain stimuli significantly activated CaMKIIα-positive neurons in the PVN and projected to the LSV in an anterograde manner. Optogenetic inhibition of this pathway was shown to relieve visceral pain and, conversely, induce visceral pain (Li et al., 2023), thus indicating that the PVN not only receives projections from the BNST but also projects to the LSV, and plays a vital role in the regulation of visceral pain.

## 4. Brain mechanisms underlying the regulatory effect of acupuncture on visceral pain

### 4.1. The supraspinal central structure involved in the regulatory effect of acupuncture on visceral pain

Acupuncture can induce analgesic effects by integrating and interacting input signals from acupuncture points and the sites of pain in the central nervous system. The visceral pain transmission pathway involves multiple central structures, including the solitary tract nucleus, hypothalamus, and the cerebral cortex. Correspondingly, signals induced by acupuncture can modulate multiple links in the transmission pathway, thus inducing analgesic effects.

#### 4.1.1. The NTS

The NTS is a critical nucleus in the medulla oblongata. Neurons in the NTS that respond to both CRD testing and electroacupuncture (EA) conditioning stimuli are considered as somatic visceral convergence neurons. EA stimulation has been shown to inhibit some CRD excitatory neurons, thereby mediating analgesic effects (Liu et al., 2014). Gastric distension has been shown to significantly induce the expression of NTS c-Fos (Liu et al., 2004). A model of visceral pain induced by the intraperitoneal injection of acetic acid was found to exhibit increased expression levels of c-fos in the NTS. In contrast, the frequency of writhing and the expression levels of c-Fos in the NTS were significantly reduced following EA at the Sibai (ST2) acupoint in rats, thus indicating that EA can significantly reduce neuronal activity of the solitary tract nucleus, thus indicating that the somatic input information generated by EA and the sensory input information induced by nociceptive stimulation of the visceral organs converge and integrate with the NTS, thus resulting in an analgesic effect (Liu et al., 2011). EA at the Zusanli (ST36) acupoint has been shown to inhibit the visceral somatic reflex caused by intense stimulation of the abdominal vagus nerve in conscious rabbits. Electrolytic damage to the NTS significantly increased the threshold for visceral pain and weaken the inhibitory effect of EA, thus indicating that the NTS plays an essential role in inhibiting the visceral pain response by acupuncture (Huang et al., 1991).

#### 4.1.2. The raphe nuclei

The raphe nuclei (RN) are composed of multiple nuclei located in the narrow area of the raphe in the brainstem. Previous research demonstrated that acupuncture can inhibit visceral somatic reflex activity in animal models, while damage to the median medulla of the raphe magnus nucleus can significantly reduce the inhibitory effect of acupuncture, thus indicating that acupuncture activates the raphe magnus nucleus and participates in the downward inhibitory effect of the visceral pain response (Du and Chao, 1976). In addition, research has shown that the raphe nucleus contains a large number of 5-HT neurons and that acupuncture can alleviate visceral hyperalgesia by mediating the activity of 5-HT neurons in the RN (Wu et al., 2010; Li et al., 2014). As an essential component of the RVM, the nucleus raphe magnus and its adjacent reticular structure was shown to cause a significant increase c-Fos protein and NR1 receptor-positive neurons in chronic visceral pain-sensitive rats with IBS. However, after EA on both sides of the Zusanli (ST36) and Shangjuxu (ST37) acupoints, the pain threshold pressure induced by CRD significantly increased, the abdominal withdrawal reflex score significantly decreased, and the number of positive neurons in the nucleus raphe magnus decreased, thus suggesting that EA can significantly inhibit an abnormal increase in the excitability of visceral responsive neurons in the RVM of a rat model of IBS, thus alleviating chronic visceral pain sensitivity (Qi and Li, 2012).

#### 4.1.3. The subnucleus reticularis dorsalis

The subnucleus reticularis dorsalis (SRD) is a nucleus located from the ventral to the dorsal side of the caudal medulla; neurons on the SRD can be specifically activate by systemic nociceptive stimuli. Ectopic nociceptive information converges on SRD neurons to play an important role in transmitting and regulating somatic and visceral nociceptive information. Yu et al. (2018) investigated the response characteristics of SRD neurons to different intensities of EA at the Zusanli (ST36) and Shangjuxu (ST37) acupoints before and after nociceptive CRD. Under normal conditions, EA can activate spontaneous activity in SRD neurons with a current range of 2–8 mA. However, during CRD, 2–8 mA of EA stimulation was shown to inhibit the increased discharge of SRD neurons caused by nociceptive CRD stimulation. It is widely considered that EA can inhibit the transmission of visceral nociceptive sensation via the interaction of body organs at SRD neurons.

#### 4.1.4. The thalamus

The thalamus receives many visceral impulses and represents the integrated center of somatic and visceral sensations. Most of the nuclei in the thalamus are closely related to visceral pain. At the level of thalamic neurons, high-frequency EA stimulation at the Zusanli (ST36) acupoint was shown to effectively inhibit the visceral nociception induced by CRD (Zhang et al., 2009). More specifically, studies have discovered that there are both somatic and visceral nociceptive neurons in the posterior nucleus of the thalamus. EA at the ST36 acupoint in the cat was shown to inhibit its nociceptive neurons; this may represent a possible mechanism for acupuncture-induced analgesic effects on visceral pain (Guoxi, 1991). Another study suggested that the visceral pain inhibitory effect of EA arises from the joint participation and coordination of two types of neurons in the ventral posterior lateral nucleus (VPL) of the thalamus. Both somatic and visceral nociceptive stimuli can simultaneously cause an increase in the discharge of excitatory pain neurons and a decrease in the discharge of inhibitory pain neurons in the VPL in rats. The injection of morphine and EA at the Zusanli (ST36) acupoint was shown to reduce discharge in the excitatory pain neurons, enhance discharge in the inhibitory pain neurons, and thus exert analgesic effects (Sun et al., 1991). In addition, visceral nociceptive stimulation can make VPL neurons more sensitive to EA stimulation in the skin receptive field; this is beneficial for the input of surface EA stimulation (Rong et al., 2015). Similarly, the nucleus medialis dorsalis (MD) also receives a large number of visceral impulses. EA at the Zusanli (ST36) acupoint was shown to significantly inhibit the discharge frequency of excited pain neurons and increase the discharge frequency of inhibitory pain neurons in the MD neurons of rats suffering from visceral pain, thus suggesting that the MD is not only involved in the transmission of visceral pain information but also in the transmission of information generated by acupuncture. These two types of incoming information interact within the MD, thus causing suppressing pain signals and inducing analgesic effects. Nevertheless, whether this analgesic effect acts in a direct or indirect manner, and the precise identity of the channels involved, requires further research (Yan et al., 2003).

#### 4.1.5. The hypothalamus

Relevant nuclei in the hypothalamus represent another central pathway involved in the effects of acupuncture treatment on visceral pain sensitivity, which may be related to changes in hypothalamic neurotransmitters and the levels of key hormones levels. For example, experimental rats with gastric distension and pain after EA exhibited a reduced stress response and increased expression levels of SP and β-EP (Yu et al., 2008; Lin et al., 2009). EA at the Shangjuxu (ST37) acupoint has been shown to significantly reduce visceral hypersensitivity in rats and upregulate CRH protein and mRNA levels in the hypothalamus (Liu et al., 2015). As one of the most essential nuclei in the anterior hypothalamus, the PVN plays an important role in regulating visceral pain and is also a central nucleus involved in the effects of acupuncture treatment on visceral pain. For example, EA at the Renzhong (DU26) and Chengjiang (CV24) acupoints has been shown to significantly inhibit the writhing response in rats caused by injecting potassium antimony tartrate. Furthermore, electrical stimulation of the PVN was shown to enhance the inhibitory effect of EA on visceral pain, while damage to the PVN disappeared. Collectively, these results suggested that EA stimulation may alleviate visceral pain by stimulating neurons in the PVN (Gong et al., 1992).

#### 4.1.6. The cerebral cortex

Stimulating the visceral nerves of cats can create a model of abdominal visceral pain; in this condition, nociceptive visceral afferent impulses and Neiguan afferent signals can converge in the visceral pain excitation unit of the cortex. The interaction between these two signals in the cortex has been shown to reduce the inhibitory convergence of visceral pain discharge and increase the facilitation convergence. EA can exert a significant inhibitory effect in the inhibitory convergence unit, thus suggesting that inhibitory convergence may represent the mechanism by which EA can suppress visceral pain. The PFC and ACC, as key nuclei of the cerebral cortex, have been shown to reduce the expression of P2X3 receptors (Weng et al., 2015) and N-methyl-D-aspartate receptors (NMDAR) in the PFC and ACC in a rat model of visceral pain following EA at the Tianshu (ST25) and Shangjuxu (ST37) acupoints, this alleviating the visceral pain response (Yu et al., 2008; Tan et al., 2019). In addition to pain, EA is also very important in mediating the processing of pain-related negative emotional information in the cerebral cortex. For example, acupuncture at the Dachangshu (BL 25) acupoint can alleviate depression anxiety-like behavior in a mouse model of visceral pain by regulating the expression of P2Y12 receptors in the PFC (Li et al., 2021) and cannabinoid receptors in the hippocampus (Hu et al., 2022).

### 4.2. Acupuncture mediates the regulation of visceral pain by neurotransmitters and receptors in specific regions of the brain

The effect of acupuncture on visceral pain depends upon interactions between the brainstem, thalamus, hypothalamus, and some advanced centers in the cerebral cortex; many neurotransmitters and receptors are involved in this analgesic mechanism.

#### 4.2.1. 5-HT

5-HT, also known as serotonin, acts as a brain-gut peptide and exerts dual effects involving neurotransmitters and hormones that can affect visceral sensation, movement, and secretion via brain-gut interaction. The raphe nucleus contains a large number of 5-HT neurons, and its 5-HT and 5-HT1a receptors can participate in visceral pain induced by different factors, often serving as important targets for the treatment of visceral pain. In an animal model of neonatal mother-infant separation stress, EA at the Zusanli (ST36) acupoint was shown to significantly increase the pain threshold of colorectal balloon dilation and reduced visceral motor response. Pain relief was related to a significant reduction in 5-HTergic activity and the expression of c-Fos in the spinal cord of the brain stem and the dorsal raphe nucleus. This indicated that EA may alleviate visceral hyperalgesia in a rat model of IBS in a manner that is potentially mediated by the downregulation of 5-HTergic activity in the central nervous system (Wu et al., 2010). 5-HT1a receptors are a class of inhibitory receptors. In a model of visceral pain induced by CRD, EA of the ear led to a significant upregulation in mRNA expression levels of the 5-HT1a receptor in both the colon and the raphe nucleus (Li et al., 2014). Collectively, this information suggests that acupuncture can alleviate visceral pain by regulating the expression of 5-HT and its receptors in the peripheral and central regions.

#### 4.2.2. Corticotropin-releasing hormone

Previous studies have shown that CRH is highly expressed in the hypothalamus of a rat model of IBS. Following EA at the Shangjuxu (ST37) acupoint, the visceral hypersensitivity response of experimental rats was significantly reduced; this coincided with a reduction in the expression of CRH and receptors in the hypothalamus (Chao et al., 2010; Liu et al., 2015). The intravenous injection of CRH receptor antagonists has been shown to alleviate the visceral hypersensitivity response in rats (Taché et al., 2004). These data indicated that CRH and its receptors are closely related to visceral pain, and that EA may exert analgesic effects by reducing the expression of CRH and its receptors in the hypothalamus. Similarly, in a rat model of IBS experiencing chronic unpredictable mild stress, EA treatment was shown to reduce the expression of both corticotropin-releasing factor (CRF) and CRF type 1 receptors in the hypothalamus, alleviate anxiety and depression, and reduce the expression of CRF type 1 receptors in the gastrointestinal mucosa, thus regulating tight junctions to repair the intestinal mucosal barrier. These data suggested that EA may mediate CRF while alleviating gastrointestinal and psychological symptoms in IBS (Chen et al., 2019).

#### 4.2.3. Glutamate

Glutamate is the most abundant excitatory neurotransmitter in the central nervous system. Excitatory toxicity has been shown to occur in glutamate neurons when glutamate is excessively released under certain abnormal conditions. N-methyl-D-aspartate (NMDA) is a gated ion channel type of glutamate receptor that is distributed widely in the brain. An increase in extracellular glutamate can lead to the activation of NMDA, thereby persistently stimulating visceral sensory neurons, promoting the expression of pain-related factors, and inducing visceral pain. The excitatory amino acid transporter system is the primary transport mechanism used to clear extracellular glutamate in the central nervous system. Removing extracellular glutamate has been shown to alleviate the excessive excitation of visceral sensory neurons caused by activation of the NMDAR. In a previous study, the levels of glutamate in the PFC of rats with IBD induced by TNBS were significantly increased; this change was reversed following EA treatment. This process is known to be related to increased levels of glutamate transporter excitatory amino acid transporter 2 in the PFC and the reduced expression levels of the NMDAR (Jiang et al., 2023). In summary, this study suggested that EA therapy can alleviate visceral pain by regulating levels of glutamate in the PFC. Another animal experiment yielded similar results, thus suggesting that EA has an excellent therapeutic effect on chronic visceral pain sensitivity in rats with IBS rats; the mechanism of action may involve the reduction of NMDAR1 expression (a positive neuron number and integrated optical density) in the RVM (Qi and Li, 2011).

#### 4.2.4. Cannabinoid receptors

Previously, researchers have tended to focus mostly on the type 1/2 cannabinoid receptor (CB1R/CB2R). CB1 is known to be involved in analgesic mechanisms in the central nervous system; γ-aminobutyric acid (GABA) and glutamate acid (Glu) can inhibit the release of neurotransmitters at the synaptic terminals of neurons. In a previous study, TNBS treatment was shown to reduce CB1R immunoreactivity in the ileum and CNS (such as ACC, PAG, and NTS), while EA reversed this trend. EA may improve visceral hypersensitivity by regulating the nuclear/regional endogenous cannabinoid system associated with visceral and descending pain regulation systems (Ma et al., 2023). In contrast, other researchers have found that EA inhibits visceral pain and IBD-induced anxiety by reducing CB1R in the ventral hippocampus (vHPC). Specifically, EA was shown to alleviate visceral hyperalgesia and anxiety in a TNBS-treated mouse model of IBD; EA reversed the overexpression of CB1R in a mouse model of IBD and reduced the expression of GABAergic neurons in the CB1R in the vHPC. The ablation of CB1R in GABAergic neurons in the vHPC alleviated anxiety in mice and simulated the anti-anxiety effect of EA. The ablation of CB1R in glutamatergic neurons in vHPC can induce severe anxiety in wild-type mice and inhibit the anti-anxiety effect of EA. However, the ablation of CB1R in vHPC neurons did not induce any alterations in visceral pain. These data indicated that the CB1R in GABAergic neurons and glutamatergic neurons are involved in the inhibitory effect of EA on anxiety in a mouse model of IBD rather than visceral pain. Collectively, these data confirm that EA can exert anti-anxiety effects by downregulating GABAergic neurons in the vHPC and by activating the CB1R in glutamate neurons (Hu et al., 2022).

#### 4.2.5. Neuropeptide

Endogenous opioid peptides are a major form of analgesic transmitter in the body and are widely distributed within the central nervous system. For example, enkephalin (ENK) and beta-endorphin (β-EP) are known to inhibit the transmission of pain information and exert analgesic effects. Previous researchers found that EA could mediate the synthesis and release of central neuropeptides, thereby alleviating visceral hyperalgesia. For example, in animal models of dysmenorrhea, the administration of EA at the Sanyinjiao (SP6) and Xuanzhong (GB39) acupoints led to the significant release of opioid peptides in the PAG; however, the Xuanzhong (GB39) acupoint was not as efficient as the Sanyinjiao (SP6) acupoint in terms of opioid release, thus indicating that EA can alleviate dysmenorrhea-like reactions in rats and achieve analgesic effects by regulating opioid peptides in the central pain modulation system. However, the effect of different acupoints on the regulation of central analgesic substances can vary (Ren et al., 2012). In the rat model of IBS-related visceral pain, EA at the Tianshu (ST25) and Shangjuxu (ST37) acupoints led to significant reduction in the AWR score. Furthermore, levels of β-EP and ENK in the hypothalamus increased significantly, thus suggesting that EA can alleviate visceral pain effectively in rats with IBS. The mechanism of action mechanism may be related to the regulation of β-EP and ENK in both the serum and hypothalamus (Ji and Huang, 2020). These findings were also confirmed by another study (Yu et al., 2008) who also suggested that acupuncture may enhance the levels of β-EP in the hypothalamus, thus inducing analgesic effects. The levels of β-EP in the hypothalamus in a mouse model of visceral pain were also significantly higher than in the normal control group, thus indicating that inflammatory pain itself may activate the central system to regulate the pain response and hyperalgesia. Acupuncture increases the levels of β-EP and also the resistance to pain (Yu et al., 2008).

In addition, substance P (SP) is an excitatory transmitter released from nociceptive afferent terminals and is closely related to pain. SP can transmit pain information, produce pain, and exert analgesic effects. Previous research found that the levels of SP and β-EP were increased in the hypothalamus of rats suffering from gastric distension pain. These results suggest that the stress caused by pain resulted in the increased secretion of SP and β-EP and that this mechanism is involved in the modulation of pain. Following low-frequency (2 Hz) and high-frequency (100 Hz) EA stimulation at the Zusanli (ST36) acupoint, the levels of SP and β-EP continued to increase significantly, thus suggesting that different frequencies of EA can lead to a significant increase in the levels of SP and β-EP, thus enhancing the analgesic effect post-release (Lin et al., 2009).

#### 4.2.6. P2 receptors

P2 receptors are a class of nucleotide and derivative receptors that can be sub-divided into the P2X type (an ion channel receptor) and the P2Y type (a G protein-coupled receptor). Adenosine triphosphate (ATP) regulates the transduction of visceral pain signals by binding to P2X and P2Y receptors. Geoffrey Burnstock was the first to discover these purinergic receptors and demonstrated experimentally that ATP was released when the epithelial cells of tubular and cystic organs were stimulated by expansion. This stimulation acted on the purinergic receptors (P2X2/3) in the subepithelial plexus to transmit pain signals to the pain center. Many subsequent studies have confirmed that EA can regulate the expression of purinergic receptors in both the peripheral and central pathways of visceral pain transmission, thereby alleviating visceral hypersensitivity in rat models with IBS, including regulatory effects on the peripheral enteric nervous system, dorsal root ganglia in the central nervous system, and the spinal cord. In a previous study, Weng et al. generated an animal model of IBS visceral pain by colorectal balloon dilation and investigated the relationship between the P2X receptor and IBS visceral hypersensitivity at different levels of the brain-gut axis (Weng et al., 2015). These researchers found that in addition to the colonic myenteric plexus, spinal cord and dorsal root ganglia, the expression of the P2X receptor in the model group was significantly higher in the PFC and ACC than in the blank group. Furthermore, the pain sensitivity of rats was reduced following EA treatment; expression levels of the P2X3 receptor in the ACC of the prefrontal cortex in the model group was also reduced, thus suggesting that EA is involved in regulating visceral pain in IBS via the central pathway and the P2X3 receptor.

Only limited research effort has focused on visceral pain and the P2Y receptor. The P2Y12 receptor is a classical G protein-coupled receptor that is expressed at high levels on the surface of activated membranes on microglial cells. Activated microglia secrete multiple proinflammatory factors to induce pain. On this basis, Li et al. investigated whether the expression levels of P2Y12 in the medial PFC (mPFC) were associated with the comorbidity associated with visceral pain and depression in IBD, and also whether EA could be used to treat IBD by targeting P2Y12 receptors (Li et al., 2021). These researchers found that TNBS-induced IBD mice exhibited visceral pain and depression and that these conditions were associated with the increased expression of P2Y12 in the mPFC. Furthermore, the administration of P2Y12 shRNA could significantly reduce visceral pain and depression in IBD mice and significantly down-regulate the levels of interleukin-1beta in the mPFC of mice, thus inhibiting microglial activation. Furthermore, EA exerted an effect that was similar to P2Y12 shRNA in that it caused a significant downregulation in the expression levels of P2Y12, attenuated microglia activation, and subsequently inhibited the levels of interleukin-1beta in the mPFC, thus alleviating visceral pain and depression in IBD mice. These data suggested that P2Y12 receptors in the mPFC may represent a new target for EA in the treatment of visceral pain and depression in IBD.

### 4.3. Acupuncture regulates functional activities of visceral pain in the brain

The rapid development of neuroimaging technology has allowed us to investigate the central mechanisms responsible for the effect of acupuncture on visceral pain. For example, resting state magnetic resonance imaging, as a non-invasive technique for investigating the structure and function of the brain, can analyze the strength of FC between a given brain region and other brain regions from the perspective of functional integration. Previous studies have shown that abnormalities in the FC of the brain may represent the basis of visceral pain and that acupuncture can exert analgesic effects by regulating abnormal functional networks in the brain. Previous research showed that following acupuncture treatment, the FC of the attention network (the temporal lobe) and the visceral pain network (such as the insula and middle cingulate gyrus) in IBS patients was weakened, the FC of the right anterior cingulate gyrus cerebellar vermis was enhanced, and that the FC of the left amygdala left posterior central gyrus was weakened. Researchers have hypothesized that acupuncture could improve the clinical symptoms of pain and anxiety by regulating the activities of FC in relevant brain regions (Zhang et al., 2022). Similarly, a previous fMRI study demonstrated brain imaging changes in D-IBS patients during rectal balloon expansion before and after moxibustion. These researchers found that moxibustion could improve the symptoms and quality-of-life of D-IBS patients and reduce the sensitivity of the rectum; this effects may be related to de-activation in the PFC and ACC (Zhu et al., 2014). In addition, glucose is the main source of energy required by the brain. Neuronal function and activity can both cause changes in the metabolism of glucose in the brain. Previously, researchers investigated changes in the local cerebral glucose metabolic rate following EA stimulation of the splanchnic nerve in a rat model. Analysis revealed significant differences in multiple brain structures, including the LC, raphe magnus, reticular giant cell nucleus, periaqueductal gray, and lateral habenular nucleus of the thalamus between the EA group and the pain group, thus indicating that these local brain structures may represent the key nuclei responsible for the effects of EA on splanchnic pain (Shu et al., 1994).

## 5. Discussion

Visceral pain is a complex and heterogeneous disease; the precise mechanisms involved have yet to be fully elucidated. Previous studies of the central mechanisms regulating pain signaling have focused on the spinal cord or the spinal dorsal horn. The rapid development of brain imaging and tracking technology has provided a new understanding of the supraspinal regulating mechanisms in visceral pain. By utilizing different animal models of visceral pain, researchers have concluded that acupuncture information and visceral nociceptive signals can reach different brain regions at the same time and create an analgesic effect by influencing the secretion and release of neurotransmitters and receptors in different brain regions. In addition, acupuncture has also been shown to regulate visceral pain by influencing functional activities and glucose metabolism in the brain. Thus, ongoing research has significantly enhanced our understanding of the brain mechanisms visceral pain and the role of acupuncture; however, some limitations and deficiencies remain that need to be considered and investigated in future research.

### 5.1. Animal models of visceral pain

Due to the inherent limitations of the previous technologies utilized in brain research, most mechanistic research focusing on neural circuits needed to be performed using appropriate animal models. At present, there are many different types of models for studying visceral pain. These models are generated in many different ways and can meet the demands of modern scientific research (Regmi and Shah, 2020). However, there is still considerable scope for improvement; for example, the application and generation of these models need to be simpler, more stable and easier to control. Furthermore, there is no objectively recognized standard for evaluating visceral pain models. We also need to consider that when different factors induce animal models of visceral pain or visceral hypersensitivity, it is highly likely that the brain regions and neural circuits responsible for coding pain signals will also differ. In addition, due to the inherent limitations of animal models, there is no accurate means of quantitatively investigating negative emotions, cognition and other reactions of visceral pain, which led to only a scant body of research relating to the regulation of visceral pain, emotion, cognition, and other effects. Therefore, the relevant models of visceral pain need to be further unified and improved. It is also vital that we develop and validate standardized evaluation criteria for different types of models.

### 5.2. Brain nuclei and circuits related to visceral pain

**Figure 1:**
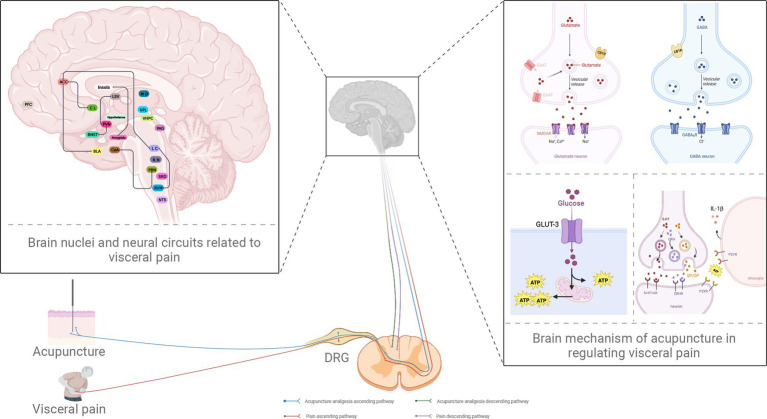
Potential neural circuits underlying visceral pain and the role of acupuncture in analgesia. PBN, parabrachial nucleus; CeA, central amygdala; PAG, periaqueductal gray; RVM, rostral ventromedial medulla; LC, locus coeruleus; ACC, anterior cingulate cortex; BNST, bed nucleus of stria terminalis; PVN, paraventricular nucleus of hypothalamus; LSV, ventral lateral septum; PFC, prefrontal cortex; BLA, basolateral amygdala; CL, claustrum; NTS, the nucleus of the solitary tract; RN, raphe nuclei; vHPC, ventral hippocampus; VPL, ventral posterior lateral nucleus; SRD, subnucleus reticularis dorsalis; MD, the nucleus medialis dorsalis; DRG, dorsal root ganglion; GLUT3, glucose transporter 3; ATP, adenosine triphosphate; EAAT, excitatory amino acid transporter; CB1R, the type 1 cannabinoid receptor; GABA, γ-aminobutyric acid; GABAAR, GABA type A receptor; NMDAR, N-methyl-d-aspartate receptor; 5-HT, 5-hydroxytryptamine; 5-HT1AR, 5-HT1A receptor; IL-1β, interleukin-1beta; P2XR, P2X receptor; P2YR, P2Y receptor; CRH, corticotropin-releasing hormone; CRHR, CRH receptor; SP, substance P; OP, opioid peptides (created with BioRender.com).

Brain dysfunction plays an essential role in the occurrence and development of visceral pain. Many brain regions are known to be related to visceral pain, including the PBN, LC, PFC, ACC, amygdala, PAG, and RVM. Furthermore, the brain nuclei form complex and delicate neural networks with each other to mediate visceral pain; typical examples include the PBN-CeA, LC-RVM, BNST-PVN, and PVN-LSV (Figure 1). However, research has indicated that the same nucleus or circuit may exhibit significant heterogeneity in the modulation of visceral pain; furthermore, different or even opposing behavioral effects may arise. For example, the LC is traditionally regarded as an inhibition center for pain. However, the LC can also project ascending fibers to multiple brain regions to promote visceral pain, thus shifting its role in pain from inhibition to promotion. This mechanism may be related to the heterogeneity of the nuclei in the LC (such as the presence of multiple subregions in the nuclei and/or the balance between different types of neurons) and the specificity of the projections upstream and downstream of each nucleus. However, although visceral pain involves many brain regions and neural circuits, this type of pain is mainly limited to the upper and lower projections between the two brain nuclei; little is known about integrating multiple brain nuclei in visceral pain.

### 5.3. The brain mechanisms responsible for the analgesic effects of acupuncture

Acupuncture is a form of treatment that involves multiple pathways and targets. Investigations of the differential mechanisms and phenomena associated with visceral pain have identified specific targets for acupuncture in terms of analgesia. Acupuncture has been shown to relieve visceral pain, depression, anxiety, and other negative emotions by regulating the functional activities of certain brain regions, including the regulation of receptor expression and neurotransmitter release, including serotonin and epinephrine (Figure 1). However, current research on the mechanisms of acupuncture in the treatment of visceral pain is limited to a single nucleus. Western studies have tended to focus on multiple brain regions and multiple circuits; however, research involving acupuncture has only focused on a small number of brain regions thus far. When considering acupuncture specifically, our knowledge of brain circuits far exceeds that of neural circuits. Little is known about whether acupuncture can act on specific neural circuits to play a role in analgesia. We hypothesize that the mechanisms underlying visceral pain are complex. It is evident that little is known about the specific mechanisms of visceral pain and the brain circuits involved. Furthermore, few researchers have focused on such mechanisms concerning the analgesic effects of acupuncture. Therefore, our future research should focus on investigating the specific mechanisms underlying the specific effects of acupuncture on visceral pain based on different brain nerve circuits. It is crucial, however, to consider the limitations of animal models in such research. It is challenging to generate animal models to analyze visceral pain; applying findings to the human condition is also very difficult. Future research needs to pay more attention to the clinical transformation of animal experiments so that we can inform clinical decision-making for the precise mechanisms underlying the effect of acupuncture on visceral pain. We must continue to apply animal models but increasingly incorporate fMRI, functional calcium imaging, and other technologies to carry out basic and clinical research to identify new brain nuclei and neural circuits. Such research will enhance our understanding of the potential central mechanisms responsible for the effect of acupuncture on visceral pain.

## Author contributions

ZD and NS searched literature, collected data, and wrote the article. ZZ, AM, LX, JZ, SS, and YL assisted in literature research and figure preparation. MH and ZL designed the research plan, guided writing, and modified the language. All authors contributed to the article and approved the submitted version.

## References

[ref1] Allen H. N. Chaudhry S. Hong V. M. Lewter L. A. Sinha G. P. Carrasquillo Y.. (2023). A parabrachial-to-amygdala circuit that determines hemispheric lateralization of somatosensory processing. Biol. Psychiatry 93, 370–381. doi: 10.1016/j.biopsych.2022.09.010 36473754 PMC9852076

[ref2] Arango-Dávila C. A. Rincón-Hoyos H. G. (2018). Depressive disorder, anxiety disorder and chronic pain: multiple manifestations of a common clinical and pathophysiological core. Rev. Colomb. Psiquiatr. (Engl. Ed). 47, 46–55. doi: 10.1016/j.rcp.2016.10.007, PMID: 29428122

[ref3] Bäckryd E. Alföldi P. (2023). Chronic pain and its relationship with anxiety and depression. Lakartidningen 120:23010 37291900

[ref4] Bai Y. Chen Y. B. Qiu X. T. Chen Y. B. Ma L. T. Li Y. Q.. (2019). Nucleus tractus solitarius mediates hyperalgesia induced by chronic pancreatitis in rats. World J. Gastroenterol. 25, 6077–6093. doi: 10.3748/wjg.v25.i40.6077, PMID: 31686764 PMC6824279

[ref5] Benarroch E. E. (2018). Locus coeruleus. Cell Tissue Res. 373, 221–232. doi: 10.1007/s00441-017-2649-1 28687925

[ref6] Berman S. Munakata J. Naliboff B. D. Chang L. Mandelkern M. Silverman D.. (2000). Gender differences in regional brain response to visceral pressure in Ibs patients. Eur. J. Pain 4, 157–172. doi: 10.1053/eujp.2000.0167, PMID: 10957697

[ref7] Bonaz B. Rivière P. J. Sinniger V. Pascaud X. Junien J. L. Fournet J.. (2000). Fedotozine, a kappa-opioid agonist, prevents spinal and supra-spinal fos expression induced by a noxious visceral stimulus in the rat. Neurogastroenterol. Motil. 12, 135–147. doi: 10.1046/j.1365-2982.2000.00188.x, PMID: 10771495

[ref8] Bouwense S. A. De Vries M. Schreuder L. T. Olesen S. S. Frøkjær J. B. Drewes A. M.. (2015). Systematic mechanism-orientated approach to chronic pancreatitis pain. World J. Gastroenterol. 21, 47–59. doi: 10.3748/wjg.v21.i1.47 25574079 PMC4284360

[ref9] Cao B. Wang J. Mu L. Poon D. C. Li Y. (2016). Impairment of decision making associated with disruption of phase-locking in the anterior cingulate cortex in viscerally hypersensitive rats. Exp. Neurol. 286, 21–31. doi: 10.1016/j.expneurol.2016.09.010, PMID: 27664369

[ref10] Cao Z. Wu X. Chen S. Fan J. Zhang R. Owyang C.. (2008). Anterior cingulate cortex modulates visceral pain as measured by visceromotor responses in viscerally hypersensitive rats. Gastroenterology 134, 535–543. doi: 10.1053/j.gastro.2007.11.057, PMID: 18242219

[ref11] Chang L. (2005). Brain responses to visceral and somatic stimuli in irritable bowel syndrome: a central nervous system disorder? Gastroenterol. Clin. N. Am. 34, 271–279. doi: 10.1016/j.gtc.2005.02.003, PMID: 15862935

[ref12] Chao G. Lv B. Meng L. Zhang S. Zahng L. Guo Y. (2010). Influence of tongxie prescription on CRF expression in spinal cord and brain of hypersensitive viscera rats. Zhongguo Zhong Yao Za Zhi 35, 2012–2016. doi: 10.4268/cjcmm20101522, PMID: 20931858

[ref13] Chen C. H. Tzeng C. R. Wang P. H. Liu W. M. Chang H. Y. Chen H. H.. (2018). Dual triggering with GnRH agonist plus hCG versus triggering with hCG alone for IVF/ICSI outcome in GnRH antagonist cycles: a systematic review and meta-analysis. Arch. Gynecol. Obstet. 298, 17–26. doi: 10.1007/s00404-018-4751-3 29600322

[ref14] Chen Y. Zhao Y. Luo D. N. Zheng H. Li Y. Zhou S. Y. (2019). Electroacupuncture regulates disorders of gut-brain interaction by decreasing corticotropin-releasing factor in a rat model of IBS. Gastroenterol. Res. Pract. 2019:1759842. doi: 10.1155/2019/1759842 31737064 PMC6815621

[ref15] Clauw D. J. Essex M. N. Pitman V. Jones K. D. (2019). Reframing chronic pain as a disease, not a symptom: rationale and implications for pain management. Postgrad. Med. 131, 185–198. doi: 10.1080/00325481.2019.1574403 30700198

[ref16] Crock L. W. Kolber B. J. Morgan C. D. Sadler K. E. Vogt S. K. Bruchas M. R.. (2012). Central amygdala metabotropic glutamate receptor 5 in the modulation of visceral pain. J. Neurosci. 32, 14217–14226. doi: 10.1523/JNEUROSCI.1473-12.2012, PMID: 23055491 PMC3494864

[ref17] Deberry J. J. Robbins M. T. Ness T. J. (2015). The amygdala central nucleus is required for acute stress-induced bladder hyperalgesia in a rat visceral pain model. Brain Res. 1606, 77–85. doi: 10.1016/j.brainres.2015.01.008 25698616 PMC4388818

[ref18] Du H. J. Chao Y. F. (1976). Localization of central structures involved in descending inhibitory effect of acupuncture on viscero-somatic reflex discharges. Sci. Sinica 19, 137–148. 1273572

[ref19] Elsenbruch S. Schmid J. Kullmann J. S. Kattoor J. Theysohn N. Forsting M.. (2014). Visceral sensitivity correlates with decreased regional gray matter volume in healthy volunteers: a voxel-based morphometry study. Pain 155, 244–249. doi: 10.1016/j.pain.2013.09.027 24099953

[ref20] Emch G. S. Hermann G. E. Rogers R. C. (2000). Tnf-alpha activates solitary nucleus neurons responsive to gastric distension. Am. J. Physiol. Gastrointest. Liver Physiol. 279, G582–G586. doi: 10.1152/ajpgi.2000.279.3.G582, PMID: 10960358

[ref21] Fan Y. Bao C. Wei Y. Wu J. Zhao Y. Zeng X.. (2020). Altered functional connectivity of the amygdala in crohn's disease. Brain Imaging Behav. 14, 2097–2106. doi: 10.1007/s11682-019-00159-8, PMID: 31628591

[ref22] Gauriau C. Bernard J. F. (2002). Pain pathways and parabrachial circuits in the rat. Exp. Physiol. 87, 251–258. doi: 10.1113/eph8702357, PMID: 11856971

[ref23] Gompf H. S. Mathai C. Fuller P. M. Wood D. A. Pedersen N. P. Saper C. B.. (2010). Locus ceruleus and anterior cingulate cortex sustain wakefulness in a novel environment. J. Neurosci. 30, 14543–14551. doi: 10.1523/JNEUROSCI.3037-10.2010, PMID: 20980612 PMC2989851

[ref24] Gong S. Yin W. P. Yin Q. Z. (1992). Involvement of vasopressinergic neurons of paraventricular nucleus in the electroacupuncture-induced inhibition of experimental visceral pain in rats. Sheng Li Xue Bao 44, 434–441. 1293759

[ref25] Graven-Nielsen T. Babenko V. Svensson P. Arendt-Nielsen L. (1998). Experimentally induced muscle pain induces hypoalgesia in heterotopic deep tissues, but not in homotopic deep tissues. Brain Res. 787, 203–210. doi: 10.1016/S0006-8993(97)01480-7, PMID: 9518613

[ref26] Grundy L. Erickson A. Brierley S. M. (2019). Visceral pain. Annu. Rev. Physiol. 81, 261–284. doi: 10.1146/annurev-physiol-020518-114525 30379615

[ref27] Guan L. Shi X. Tang Y. Yan Y. Chen L. Chen Y.. (2022). Contribution of amygdala histone acetylation in early life stress-induced visceral hypersensitivity and emotional comorbidity. Front. Neurosci. 16:843396. doi: 10.3389/fnins.2022.843396, PMID: 35600618 PMC9120649

[ref28] Guoxi T. (1991). The action of the visceronociceptive neurons in the posterior group of thalamic nuclei: possible mechanism of acupuncture analgesia on visceral pain. Kitasato Arch. Exp. Med. 64, 43–55. PMID: 1798237

[ref29] Hallock H. L. Adiraju S. Miranda-Barrientos J. Mcinerney J. M. Oh S. Debrosse A. C.. (2023). Electrophysiological correlates of attention in the locus coeruleus - anterior cingulate cortex circuit during the rodent continuous performance test. Biorxiv. doi: 10.1101/2023.04.19.537406 PMC10789747 37563281

[ref30] Han J. S. Neugebauer V. (2004). Synaptic plasticity in the amygdala in a visceral pain model in rats. Neurosci. Lett. 361, 254–257. doi: 10.1016/j.neulet.2003.12.027 15135941

[ref31] Hasan M. Lei Z. Akter M. Iqbal Z. Usaila F. Ramkrishnan A. S.. (2023). Chemogenetic activation of astrocytes promotes remyelination and restores cognitive deficits in visceral hypersensitive rats. Iscience 26:105840. doi: 10.1016/j.isci.2022.105840, PMID: 36619970 PMC9812719

[ref32] Holschneider D. P. Guo Y. Mayer E. A. Wang Z. (2016). Early life stress elicits visceral hyperalgesia and functional reorganization of pain circuits in adult rats. Neurobiol. Stress 3, 8–22. doi: 10.1016/j.ynstr.2015.12.003, PMID: 26751119 PMC4700548

[ref33] Hu X. F. Zhang H. Yu L. L. Ge W. Q. Zhan-Mu O. Y. Li Y. Z.. (2022). Electroacupuncture reduces anxiety associated with inflammatory bowel disease by acting on cannabinoid cb1 receptors in the ventral hippocampus in mice. Front. Pharmacol. 13:919553. doi: 10.3389/fphar.2022.919553, PMID: 35873560 PMC9305710

[ref34] Huang Z. Liu N. Zhong S. Lu J. Zhang N. (1991). The role of nucleus tractus solitarii (Nts) in acupuncture inhibition of visceral-somatic reflex (Vsr). Zhen Ci Yan Jiu 16, 43–47. PMID: 1873901

[ref35] Huang S. T. Song Z. J. Liu Y. Luo W. C. Yin Q. Zhang Y. M. (2021). BNST_AV_ ^GABA^-PVN^CRF^ circuit regulates visceral hypersensitivity induced by maternal separation in Vgat-Cre mice. Front. Pharmacol. 12:615202. doi: 10.3389/fphar.2021.615202, PMID: 33815103 PMC8017215

[ref36] Imbe H. Murakami S. Okamoto K. Iwai-Liao Y. Senba E. (2004). The effects of acute and chronic restraint stress on activation of erk in the rostral ventromedial medulla and locus coeruleus. Pain 112, 361–371. doi: 10.1016/j.pain.2004.09.015, PMID: 15561392

[ref37] Iqbal Z. Lei Z. Ramkrishnan A. S. Liu S. Hasan M. Akter M.. (2023). Adrenergic signalling to astrocytes in anterior cingulate cortex contributes to pain-related aversive memory in rats. Commun. Biol. 6:10. doi: 10.1038/s42003-022-04405-6, PMID: 36604595 PMC9816175

[ref38] Iqbal Z. Liu S. Lei Z. Ramkrishnan A. S. Akter M. Li Y. (2022). Astrocyte L-lactate signaling in the acc regulates visceral pain aversive memory in rats. Cells 12:26. doi: 10.3390/cells12010026 36611820 PMC9818423

[ref39] Jackson J. Smith J. B. Lee A. K. (2020). The anatomy and physiology of claustrum-cortex interactions. Annu. Rev. Neurosci. 43, 231–247. doi: 10.1146/annurev-neuro-092519-101637, PMID: 32084328

[ref40] Ji J. H. Huang Z. Z. (2020). Study On the regulating effects of electroacupuncture on Β-endorphin and enkephalin in visceral pain rats with irritable bowel syndrome. World Chinese Med., 15, 2873–2877. (In Chinese).

[ref41] Ji G. Li Z. Neugebauer V. (2015). Reactive oxygen species mediate visceral pain-related amygdala plasticity and behaviors. Pain 156, 825–836. doi: 10.1097/j.pain.0000000000000120, PMID: 25734993 PMC4402250

[ref42] Jiang H. Li R. Zhang F. Zhou F. Lin J. Kong N.. (2023). Electroacupuncture alleviates 46-trinitrobenzene sulfonic acid-induced visceral pain via the glutamatergic pathway in the prefrontal cortex. Oxidative Med. Cell. Longev. 2023:4463063. doi: 10.1155/2023/4463063 PMC9879690 36713031

[ref43] Juel J. Liguori S. Liguori A. Poulsen J. L. Valeriani M. Graversen C.. (2017). Acupuncture for pain in chronic pancreatitis: a single-blinded randomized crossover trial. Pancreas 46, 170–176. doi: 10.1097/MPA.0000000000000749, PMID: 28060186

[ref44] Kang Y. Zhao Y. Guo R. Zhang M. Wang Y. Mu Y.. (2013). Activation of erk signaling in rostral ventromedial medulla is dependent on afferent input from dorsal column pathway and contributes to acetic acid-induced visceral nociception. Neurochem. Int. 63, 389–396. doi: 10.1016/j.neuint.2013.07.005, PMID: 23876632

[ref45] Koga K. Yamada A. Song Q. Li X. H. Chen Q. Y. Liu R. H.. (2020). Ascending noradrenergic excitation from the locus coeruleus to the anterior cingulate cortex. Mol. Brain 13:49. doi: 10.1186/s13041-020-00586-5, PMID: 32216807 PMC7098117

[ref46] Kong D. Zhang Y. Gao P. Pan C. Deng H. Xu S.. (2023). The locus coeruleus input to the rostral ventromedial medulla mediates stress-induced colorectal visceral pain. Acta Neuropathol. Commun. 11:65. doi: 10.1186/s40478-023-01537-6, PMID: 37062831 PMC10108465

[ref47] Krebs R. M. Fias W. Achten E. Boehler C. N. (2013). Picture novelty attenuates semantic interference and modulates concomitant neural activity in the anterior cingulate cortex and the locus coeruleus. NeuroImage 74, 179–187. doi: 10.1016/j.neuroimage.2013.02.027, PMID: 23454569

[ref48] Li H. Hu S. Zhang J. Zhou J. Ran H. Tang Y.. (2014). Effects and mechanisms of auricular electroacupuncture on visceral pain induced by colorectal distension in conscious rats. Acupunct. Med. 32, 472–477. doi: 10.1136/acupmed-2014-010575, PMID: 25193927

[ref49] Li Y. C. Wang Q. Li M. G. Hu S. F. Xu G. Y. (2023). A paraventricular hypothalamic nucleus input to ventral of lateral septal nucleus controls chronic visceral pain. Pain 164, 625–637. doi: 10.1097/j.pain.0000000000002750 35994589 PMC9916060

[ref50] Li J. Zhang M. M. Tu K. Wang J. Feng B. Zhang Z. N.. (2015). The excitatory synaptic transmission of the nucleus of solitary tract was potentiated by chronic myocardial infarction in rats. PLoS One 10:E0118827. doi: 10.1371/journal.pone.0118827, PMID: 25756354 PMC4354907

[ref51] Li Y. Zhang H. Yang J. Zhan M. Hu X. Liu Y.. (2021). P2y12 receptor as a new target for electroacupuncture relieving comorbidity of visceral pain and depression of inflammatory bowel disease. Chin. Med. 16:139. doi: 10.1186/s13020-021-00553-9, PMID: 34930362 PMC8686637

[ref52] Liang S. H. Yin J. B. Sun Y. Bai Y. Zhou K. X. Zhao W. J.. (2016). Collateral projections from the lateral parabrachial nucleus to the paraventricular thalamic nucleus and the central amygdaloid nucleus in the rat. Neurosci. Lett. 629, 245–250. doi: 10.1016/j.neulet.2016.07.017 27423318

[ref53] Lin Y. P. Peng Y. Yi S. X. Tang S. (2009). Effect of different frequency electroacupuncture on the expression of substance P and beta-endorphin in the hypothalamus in rats with gastric distension-induced pain. Zhen Ci Yan Jiu 34, 252–257. 19916289

[ref54] Liu H. R. Fang X. Y. Wu H. G. Wu L. Y. Li J. Weng Z. J.. (2015). Effects of electroacupuncture on corticotropin-releasing hormone in rats with chronic visceral hypersensitivity. World J. Gastroenterol. 21, 7181–7190. doi: 10.3748/wjg.v21.i23.7181, PMID: 26109804 PMC4476879

[ref55] Liu J. Fu W. Yi W. Xu Z. Liao Y. Li X.. (2011). Extrasegmental analgesia of heterotopic electroacupuncture stimulation on visceral pain rats. Brain Res. 1373, 160–171. doi: 10.1016/j.brainres.2010.12.013 21163255

[ref56] Liu K. Gao X. Y. Li L. Ben H. Qin Q. G. Zhao Y. X.. (2014). Neurons in the nucleus tractus solitarius mediate the acupuncture analgesia in visceral pain rats. Auton. Neurosci. 186, 91–94. doi: 10.1016/j.autneu.2014.08.004, PMID: 25204607

[ref57] Liu J. H. Li J. Yan J. Chang X. R. Cui R. F. He J. F.. (2004). Expression of C-Fos in the nucleus of the solitary tract following electroacupuncture at facial acupoints and gastric distension in rats. Neurosci. Lett. 366, 215–219. doi: 10.1016/j.neulet.2004.05.068, PMID: 15276250

[ref58] Liu L. Tsuruoka M. Maeda M. Hayashi B. Wang X. Inoue T. (2008). Descending modulation of visceral nociceptive transmission from the locus coeruleus/subcoeruleus in the rat. Brain Res. Bull. 76, 616–625. doi: 10.1016/j.brainresbull.2008.04.010, PMID: 18598853

[ref59] Louwies T. Orock A. Greenwood-Van Meerveld B. (2021). Stress-induced visceral pain in female rats is associated with epigenetic remodeling in the central nucleus of the amygdala. Neurobiol. Stress 15:100386. doi: 10.1016/j.ynstr.2021.100386, PMID: 34584907 PMC8456109

[ref60] Lu H. J. Gao Y. J. (2023). Astrocytes in chronic pain: cellular and molecular mechanisms. Neurosci. Bull. 39, 425–439. doi: 10.1007/s12264-022-00961-3, PMID: 36376699 PMC10043112

[ref61] Lund I. Lundeberg T. (2016). Is acupuncture effective in the treatment of pain in endometriosis? J. Pain Res. 9, 157–165. doi: 10.2147/JPR.S55580, PMID: 27069371 PMC4818044

[ref62] Ma N. Li X. Li Q. Yang D. Zhuang S. Nan S.. (2023). Electroacupuncture relieves visceral hypersensitivity through modulation of the endogenous cannabinoid system. Acupunct. Med. 41, 224–234. doi: 10.1177/09645284221107699, PMID: 35957508

[ref63] Michaelides A. Zis P. (2019). Depression, anxiety and acute pain: links and management challenges. Postgrad. Med. 131, 438–444. doi: 10.1080/00325481.2019.1663705 31482756

[ref64] Murphy A. Z. Suckow S. K. Johns M. Traub R. J. (2009). Sex differences in the activation of the spinoparabrachial circuit by visceral pain. Physiol. Behav. 97, 205–212. doi: 10.1016/j.physbeh.2009.02.037, PMID: 19275905 PMC2788429

[ref65] Nagai M. Kishi K. Kato S. (2007). Insular cortex and neuropsychiatric disorders: a review of recent literature. Eur. Psychiatry 22, 387–394. doi: 10.1016/j.eurpsy.2007.02.006, PMID: 17416488

[ref66] Ntamati N. R. Acuña M. A. Nevian T. (2023). Pain-induced adaptations in the claustro-cingulate pathway. Cell Rep. 42:112506. doi: 10.1016/j.celrep.2023.112506 37182208 PMC10242445

[ref67] Orock A. Louwies T. Ligon C. O. Mohammadi E. Greenwood-Van Meerveld B. (2021). Environmental enrichment prevents stress-induced epigenetic changes in the expression of glucocorticoid receptor and corticotrophin releasing hormone in the central nucleus of the amygdala to inhibit visceral hypersensitivity. Exp. Neurol. 345:113841. doi: 10.1016/j.expneurol.2021.113841 34390704 PMC8953442

[ref68] Palmiter R. D. (2018). The parabrachial nucleus: CGRP neurons function as a general alarm. Trends Neurosci. 41, 280–293. doi: 10.1016/j.tins.2018.03.007, PMID: 29703377 PMC5929477

[ref69] Pei L. Chen H. Guo J. Chen L. Wu X. Xu W.. (2018). Effect of acupuncture and its influence on visceral hypersensitivity in Ibs-D patients: study protocol for a randomized controlled trial. Medicine (Baltimore) 97:E10877. doi: 10.1097/MD.0000000000010877, PMID: 29794793 PMC6392752

[ref70] Pusceddu M. M. Gareau M. G. (2018). Visceral pain: gut microbiota, a new hope? J. Biomed. Sci. 25:73. doi: 10.1186/s12929-018-0476-7, PMID: 30309367 PMC6182804

[ref71] Qi D. B. Li W. M. (2011). Effect of electroacupuncture on expression of NMDA-R1 receptor in the rostral ventromedia medulla of rats with chronic visceral hyperalgesia. Shanghai J. Acupunct. Moxibust., 30, 491–494. (In Chinese).

[ref72] Qi D. B. Li W. M. (2012). Effects of electroacupuncture on expression of c-fos protein and N-methyl-D-aspartate receptor 1 in the rostral ventromedial medulla of rats with chronic visceral hyperalgesia. Zhong Xi Yi Jie He Xue Bao 10, 416–423. doi: 10.3736/jcim20120410, PMID: 22500715

[ref73] Qi L. Lin S. H. Ma Q. (2023). Spinal VGLUT3 lineage neurons drive visceral mechanical allodynia but not sensitized visceromotor reflexes. Neuron 111:669–681.e5. doi: 10.1016/j.neuron.2022.12.003 36584681

[ref74] Qin M. Huang Y. X. Wang J. J. Duan L. Cao R. Rao Z. R. (2006). Effect of fluorocitrate injected into lateral ventricle of cerebrum on expression of Fos and glial fibrillary acidic protein in nucleus of solitary tract in medulla oblongata in a rat model of visceral pain. Chinese J. Anesthesiol., 26, 684–687. (In Chinese).

[ref75] Randich A. Mebane H. Deberry J. J. Ness T. J. (2008). Rostral ventral medulla modulation of the visceromotor reflex evoked by urinary bladder distension in female rats. J. Pain 9, 920–926. doi: 10.1016/j.jpain.2008.05.011, PMID: 18619908 PMC2576287

[ref76] Regmi B. Shah M. K. (2020). Possible implications of animal models for the assessment of visceral pain. Anim. Model Exp. Med. 3, 215–228. doi: 10.1002/ame2.12130, PMID: 33024943 PMC7529330

[ref77] Ren X. X. Guo M. W. Zhao Y. F. Ding X. Y. Li C. H. Ji B.. (2012). Effects of electroacupuncture on pain reaction, expression of spinal Κ-opioid receptor and contents of enkephalin and Β-endorphin in periaqueductal gray of midbain in dysmenorrhea model rats. Acupuncture Res., 37, 1–7. doi: 10.13702/j.1000-0607.2012.01.006 (In Chinese)., PMID: 22574561

[ref78] Ren D. Li J. N. Qiu X. T. Wan F. P. Wu Z. Y. Fan B. Y.. (2022). Anterior cingulate cortex mediates hyperalgesia and anxiety induced by chronic pancreatitis in rats. Neurosci. Bull. 38, 342–358. doi: 10.1007/s12264-021-00800-x, PMID: 34907496 PMC9068840

[ref79] Risold P. Y. Swanson L. W. (1997). Chemoarchitecture of the rat lateral septal nucleus. Brain Res. Brain Res. Rev. 24, 91–113. doi: 10.1016/S0165-0173(97)00008-8, PMID: 9385453

[ref80] Rong P. J. Zhao J. J. Yu L. L. Li L. Ben H. Li S. Y.. (2015). Function of nucleus ventralis posterior lateralis thalami in acupoint sensitization phenomena. Evid. Based Complement. Alternat. Med. 2015:516851. doi: 10.1155/2015/516851 26161121 PMC4487708

[ref81] Sanoja R. Tortorici V. Fernandez C. Price T. J. Cervero F. (2010). Role of RVM neurons in capsaicin-evoked visceral nociception and referred hyperalgesia. Eur. J. Pain 14:120.E1-9. doi: 10.1016/j.ejpain.2009.04.006, PMID: 19443247 PMC2914578

[ref82] Schwaber J. S. Sternini C. Brecha N. C. Rogers W. T. Card J. P. (1988). Neurons containing calcitonin gene-related peptide in the parabrachial nucleus project to the central nucleus of the amygdala. J. Comp. Neurol. 270, 398–399. 10.1002/cne.902700310 2836477

[ref83] Shu J. Li K. Y. Huang D. K. (1994). The central effect of electro-acupuncture analgesia on visceral pain of rats: a study using the [3h] 2-deoxyglucose method. Acupunct Electrother. Res. 19, 107–117. doi: 10.3727/036012994816357330, PMID: 7863835

[ref84] Šimić G. Tkalčić M. Vukić V. Mulc D. Španić E. Šagud M.. (2021). Understanding emotions: origins and roles of the amygdala. Biomol. Ther. 11:823. doi: 10.3390/biom11060823, PMID: 34072960 PMC8228195

[ref85] Smith J. B. Lee A. K. Jackson J. (2020). The claustrum. Curr. Biol. 30, R1401–R1406. doi: 10.1016/j.cub.2020.09.069 33290700

[ref86] Song Y. Meng Q. X. Wu K. Hua R. Song Z. J. Song Y.. (2020). Disinhibition of PVN-projecting gabaergic neurons in AV region in bnst participates in visceral hypersensitivity in rats. Psychoneuroendocrinology 117:104690. doi: 10.1016/j.psyneuen.2020.104690 32417623

[ref87] Sun M. Li Y. Zhang J. Bian J. (1991). Effects of noxious stimuli on the discharges of pain-excitation neurons and pain-inhibition neurons in the nucleus ventralis posterolalis of thalamus in the rat and a modulating action of electroacupuncture on its electric activities. Zhen Ci Yan Jiu 16, 19–22. PMID: 1873897

[ref88] Taché Y. Martinez V. Wang L. Million M. (2004). Crf1 receptor signaling pathways are involved in stress-related alterations of colonic function and viscerosensitivity: implications for irritable bowel syndrome. Br. J. Pharmacol. 141, 1321–1330. doi: 10.1038/sj.bjp.0705760, PMID: 15100165 PMC1574904

[ref89] Tan L. H. Li K. G. Wu Y. Y. Guo M. W. Lan Y. Wang S.. (2019). Effect of electroacupuncture at different acupoints on the expression of NMDA receptors in acc and colon in IBS rats. Evid. Based Complement. Alternat. Med. 2019:4213928. doi: 10.1155/2019/4213928 30854008 PMC6377955

[ref90] Taylor B. K. Westlund K. N. (2017). The noradrenergic locus coeruleus as a chronic pain generator. J. Neurosci. Res. 95, 1336–1346. doi: 10.1002/jnr.23956, PMID: 27685982 PMC5374049

[ref91] Todd J. Austin H. Clarke P. Notebaert L. (2022). Chronic pain, insomnia and their mutual maintenance: a call for cognitive bias research. J. Pain 23, 1530–1542. doi: 10.1016/j.jpain.2022.03.241, PMID: 35472519

[ref92] Traub R. J. Sengupta J. N. Gebhart G. F. (1996). Differential C-Fos expression in the nucleus of the solitary tract and spinal cord following noxious gastric distention in the rat. Neuroscience 74, 873–884. doi: 10.1016/0306-4522(96)00173-X, PMID: 8884783

[ref93] Tsuruoka M. Wang D. Tamaki J. Inoue T. (2010). Descending influence from the nucleus locus coeruleus/subcoeruleus on visceral nociceptive transmission in the rat spinal cord. Neuroscience 165, 1019–1024. doi: 10.1016/j.neuroscience.2009.11.055, PMID: 19958815

[ref94] Tsushima H. Zhang Y. Muratsubaki T. Kanazawa M. Fukudo S. (2021). Oxytocin antagonist induced visceral pain and corticotropin-releasing hormone neuronal activation in the central nucleus of the amygdala during colorectal distention in mice. Neurosci. Res. 168, 41–53. doi: 10.1016/j.neures.2021.04.011, PMID: 33932549

[ref95] Uddin L. Q. Nomi J. S. Hébert-Seropian B. Ghaziri J. Boucher O. (2017). Structure and function of the human insula. J. Clin. Neurophysiol. 34, 300–306. doi: 10.1097/WNP.0000000000000377 28644199 PMC6032992

[ref96] Veinante P. Yalcin I. Barrot M. (2013). The amygdala between sensation and affect: a role in pain. J. Mol. Psychiatry 1:9. doi: 10.1186/2049-9256-1-9, PMID: 25408902 PMC4223879

[ref97] Vera-Portocarrero L. P. Xie J. Y. Kowal J. Ossipov M. H. King T. Porreca F. (2006). Descending facilitation from the rostral ventromedial medulla maintains visceral pain in rats with experimental pancreatitis. Gastroenterology 130, 2155–2164. doi: 10.1053/j.gastro.2006.03.025 16762636

[ref98] Weng Z. J. Wu L. Y. Zhou C. L. Dou C. Z. Shi Y. Liu H. R.. (2015). Effect of electroacupuncture on P2x3 receptor regulation in the peripheral and central nervous systems of rats with visceral pain caused by irritable bowel syndrome. Purinergic Signal 11, 321–329. doi: 10.1007/s11302-015-9447-6, PMID: 25809868 PMC4529849

[ref99] Woelk J. Goerlitz D. Wachholtz A. (2020). I'm tired and it hurts! sleep quality and acute pain response in a chronic pain population. Sleep Med. 67, 28–32. doi: 10.1016/j.sleep.2019.10.017, PMID: 31884308 PMC6980709

[ref100] Wu K. Liu Y. Y. Shao S. Song W. Chen X. H. Dong Y. T.. (2023). The microglial innate immune receptors TREM-1 and TREM-2 in the anterior cingulate cortex (ACC) drive visceral hypersensitivity and depressive-like behaviors following dss-induced colitis. Brain Behav. Immun. 112, 96–117. doi: 10.1016/j.bbi.2023.06.003, PMID: 37286175

[ref101] Wu J. C. Ziea E. T. Lao L. Lam E. F. Chan C. S. Liang A. Y.. (2010). Effect of electroacupuncture on visceral hyperalgesia, serotonin and Fos expression in an animal model of irritable bowel syndrome. J. Neurogastroenterol. Motil. 16, 306–314. doi: 10.5056/jnm.2010.16.3.306, PMID: 20680170 PMC2912124

[ref102] Xu Q. Y. Zhang H. L. Du H. Li Y. C. Ji F. H. Li R.. (2022). Identification of a glutamatergic claustrum-anterior cingulate cortex circuit for visceral pain processing. J. Neurosci. 42, 8154–8168. doi: 10.1523/JNEUROSCI.0779-22.2022, PMID: 36100399 PMC9637003

[ref103] Xu J. N. Zhu H. W. Tang A. M. Sun J. L. Ye L. H. Zhang Y. M. (2022). Mechanism of acute visceral pain mediated by Cb1rin hypothalamic paraventricular nucleus. J. Xuzhou Med.Univ., 42, 391–395. (In Chinese).

[ref104] Yan N. Cao B. Xu J. Hao C. Zhang X. Li Y. (2012). Glutamatergic activation of anterior cingulate cortex mediates the affective component of visceral pain memory in rats. Neurobiol. Learn. Mem. 97, 156–164. doi: 10.1016/j.nlm.2011.11.003, PMID: 22107830

[ref105] Yan L. P. Ma C. Xiang X. R. He C. Liu Z. C. Wang L. L. (2003). Effect of electro - acupuncture on the pain sensitive unit firingsin mediodorsal thalamic nucleus of rats. Chinese J. Trad. Med. Sci. Technol., 10:129-130+5. (In Chinese).

[ref106] Yu W. C. Huang G. Y. Zhang M. M. (2008). Influence of connexin 43 gene knockout on the analgesic effect of acupuncture in visceral pain mice. Zhen Ci Yan Jiu 33, 3–6. 18386636

[ref107] Yu L. Li L. Qin Q. Yu Y. Cui X. Rong P.. (2018). Electroacupuncture inhibits visceral nociception via somatovisceral interaction at subnucleus reticularis dorsalis neurons in the rat medulla. Front. Neurosci. 12:775. doi: 10.3389/fnins.2018.00775, PMID: 30425615 PMC6218567

[ref108] Yuan T. Manohar K. Latorre R. Orock A. Greenwood-Van Meerveld B. (2020). Inhibition of microglial activation in the amygdala reverses stress-induced abdominal pain in the male rat. Cell. Mol. Gastroenterol. Hepatol. 10, 527–543. doi: 10.1016/j.jcmgh.2020.04.020, PMID: 32408032 PMC7394753

[ref109] Yuan T. Orock A. Greenwood-Vanmeerveld B. (2022). An enriched environment reduces chronic stress-induced visceral pain through modulating microglial activity in the central nucleus of the amygdala. Am. J. Physiol. Gastrointest. Liver Physiol. 322, G223–G233. doi: 10.1152/ajpgi.00307.2021, PMID: 34877892 PMC8793868

[ref110] Zhang W. X. Liu Y. K. Yao G. Liang Z. P. (2022). Effect of acupuncture on brain functional connectivity of resting-state fMRI in patients with diarrhea-predominant irritable bowel syndrome. Int. J. Med. Radiol., 45, 621–625. doi: 10.19300/j.2022.L19676 (In Chinese).

[ref111] Zhang G. Yu L. Chen Z. Y. Zhu J. S. Hua R. Qin X.. (2016). Activation of corticotropin-releasing factor neurons and microglia in paraventricular nucleus precipitates visceral hypersensitivity induced by colorectal distension in rats. Brain Behav. Immun. 55, 93–104. doi: 10.1016/j.bbi.2015.12.022 26743854

[ref112] Zhang J. L. Zhang S. P. Zhang H. Q. (2009). Effect of electroacupuncture on thalamic neuronal response to visceral nociception. Eur. J. Pain 13, 366–372. doi: 10.1016/j.ejpain.2008.04.016, PMID: 18547846

[ref113] Zhong X. L. Wei R. Zhou P. Luo Y. W. Wang X. Q. Duan J.. (2012). Activation of anterior cingulate cortex extracellular signal-regulated kinase-1 and −2 (Erk1/2) regulates acetic acid-induced, pain-related anxiety in adult female mice. Acta Histochem. Cytochem. 45, 219–225. doi: 10.1267/ahc.12002, PMID: 23012487 PMC3445761

[ref114] Zhu Y. Wu Z. Ma X. Liu H. Bao C. Yang L.. (2014). Brain regions involved in moxibustion-induced analgesia in irritable bowel syndrome with diarrhea: a functional magnetic resonance imaging study. BMC Complement. Altern. Med. 14:500. doi: 10.1186/1472-6882-14-500, PMID: 25516481 PMC4301658

[ref115] Zingg B. Dong H. W. Tao H. W. Zhang L. I. (2018). Input-output organization of the mouse claustrum. J. Comp. Neurol. 526, 2428–2443. doi: 10.1002/cne.24502, PMID: 30252130 PMC6196111

